# The Control Method of Autonomous Flight Avoidance Barriers of UAVs in Confined Environments

**DOI:** 10.3390/s23135896

**Published:** 2023-06-25

**Authors:** Tiantian Dong, Yonghong Zhang, Qianyu Xiao, Yi Huang

**Affiliations:** 1 School of Electronics and Information Engineering, Nanjing University of Information Science and Technology, Nanjing 210044, China; 2 School of Microelectronics, Jiangsu Vocational College of Information Technology, Wuxi 214153, China; 3 School of Applied Technology, Changzhou University, Changzhou 213164, China

**Keywords:** multi-rotor UAV, autonomous navigation, 3D-VFH, local obstacle avoidance

## Abstract

This paper proposes an improved 3D-Vector Field Histogram (3D-VFH) algorithm for autonomous flight and local obstacle avoidance of multi-rotor unmanned aerial vehicles (UAVs) in a confined environment. Firstly, the method employs a target point coordinate system based on polar coordinates to convert the point cloud data, considering that long-range point cloud information has no effect on local obstacle avoidance by UAVs. This enables UAVs to effectively utilize obstacle information for obstacle avoidance and improves the real-time performance of the algorithm. Secondly, a sliding window algorithm is used to estimate the optimal flight path of the UAV and implement obstacle avoidance control, thereby maintaining the attitude stability of the UAV during obstacle avoidance flight. Finally, experimental analysis is conducted, and the results show that the UAV has good attitude stability during obstacle avoidance flight, can autonomously follow the expected trajectory, and can avoid dynamic obstacles, achieving precise obstacle avoidance.

## 1. Introduction

In recent years, with the rapid development of emerging technologies such as the industrial internet of things, big data, and artificial intelligence, UAVs have had a broad range of applications in inspecting, detecting, and exploring dangerous or inaccessible areas. Currently, outdoor UAV applications are relatively mature [1,2,3,4], but in complex confined environments [5,6,7], especially in industrial scenarios such as high-altitude, high-dust, and double-blind conditions (no Global Positioning System (GPS) or illumination) like boiler rooms in thermal power plants, there are many technical challenges for UAVs to perform intelligent flight, high-definition imaging, and image processing. This has become one of the most important issues that urgently needs to be addressed in the industrial UAV industry [8,9,10,11,12,13,14,15]. The development of UAV obstacle avoidance control technology has engineering significance.

The confined environment with no GPS or lighting is a “double-blind environment”, in which traditional UAV positioning methods based on GPS are not applicable. Therefore, high-precision UAV positioning is one of the key technologies studied in this project. Traditional inertial positioning devices can only control the UAV’s flight attitude but cannot perceive the external environment or control the UAV to actively avoid obstacles [16,17,18,19,20,21]. To address this issue, many scholars have proposed various solutions. For example, reference [22] uses an end-to-end decision control model based on the Deep Deterministic Policy Gradient (DDPG) algorithm to achieve UAV obstacle avoidance decisions. Reference [23] avoids UAV collisions with high-speed obstacles by combining the Chance-Constraints Based on Obstacle Velocity (CCOV) method with previous location information-based opportunity constraints. The above literature introduces different obstacle avoidance algorithms based on the three-dimensional artificial potential field to achieve UAV obstacle avoidance control functions, but there is still room for improvement. This is a research paper on autonomous flight and active obstacle avoidance in a multi-rotor UAV. The translation should be both professional and idiomatic.

Therefore, this paper addresses the issue of the inaccuracy of the local obstacle avoidance function when using traditional artificial potential field methods. By equipping a 3D laser radar sensor and constructing a 3D artificial potential field, the paper improves the target point coordinate system conversion method to enhance the reliability of the UAV’s flight towards the target point [24]. Then, the sliding window algorithm is optimized to improve the accuracy of selecting avoidance directions for dynamic obstacles. Finally, the paper proposes an improved 3D-VFH obstacle avoidance algorithm and conducts simulation experiments and experimental tests. First, an experimental testing platform is built based on a multi-rotor UAV, and then autonomous flight tests and autonomous obstacle avoidance tests are conducted to evaluate the impact on the stability of the multi-rotor UAV’s motion. Finally, the stability of the multi-rotor UAV’s autonomous flight and obstacle avoidance processes with the 3D-VFH algorithm is validated, providing important guidance for autonomous flight and precise obstacle avoidance of the multi-rotor UAVs in enclosed spaces. The research is divided into four parts. The first part is related research on obstacle avoidance of unmanned aerial vehicles and artificial potential fields; the second part is the research methods section, which mainly describes the content of improving the 3D-VFH obstacle avoidance algorithm; the third part is the results section, which analyzes the relevant application effects of the 3D-VFH obstacle avoidance algorithm; and the fourth part is the conclusion section, summarizing the purpose, methods, and other related content of the study.

## 2. Related Work

Zammit C et al. used a fast search random tree for path planning of three-dimensional unmanned aerial vehicles. After comparing the results, its application effect is superior to the A* algorithm [25]. Chen H et al. proposed the taboo pyramid method to distinguish and track static obstacles and conduct simulation analysis. After verification, the proposed method has a good application effect and can reduce computational time [26]. Zhou Y et al. proposed a three-dimensional biologically inspired path planning algorithm to solve the dynamic obstacle avoidance path planning problem of unmanned aerial vehicles in unknown environmental maps. After experimental verification, this algorithm can effectively carry out relevant path planning [27]. Pan Z and others faced the obstacle avoidance control problem of multiple unmanned aerial vehicle formations and, based on PID technology combined with improved artificial potential fields, solved obstacle avoidance and collision problems. From the obtained results, it can be seen that the proposed algorithm has a good problem-solving effect [28]. Xue Z et al. faced the obstacle avoidance problem of unmanned aerial vehicles (UAVs) and, based on visual technology, trained UAVs using soft actor-critical algorithms to enable them to automatically avoid obstacles. The results show that the algorithm performs well [29].

Tang J et al. chose to improve the artificial potential field algorithm in order to plan the trajectories of multiple drones in a three-dimensional environment in order to avoid drones colliding with obstacles. After verification, this method can achieve satisfactory results [30]. Xu T et al. conducted research on path optimization by improving the artificial potential field method to improve the effectiveness of obstacle avoidance in robotic arms. Through experiments, it can be found that this method can effectively avoid obstacles [31].

In summary, in the field of drone research, the obstacle avoidance problem of drones is the focus and difficulty. Therefore, this article studies the automatic obstacle avoidance of drones in a limited environment. However, the performance of the artificial potential field in the obstacle avoidance algorithm is better, and research on three-dimensional space is still limited at present. Therefore, the article will use the 3D-VFH algorithm as aresearch method to carry out research on the control of the automatic flight avoidance barrier of UAVs.

## 3. Improved 3D-VFH Local Obstacle Avoidance Control Algorithm

The 3D-VFH algorithm is an improvement of the 2D Vector Field Histogram+ (2D-VFH+) algorithm and is designed for obstacle avoidance in 3D confined environments for UAVs [32,33]. The principle behind this algorithm is to use an octree map to describe local environmental information and obtain various information about the octree map. The obstacle information in the enclosed 3D environment is converted into a histogram through dimensionality reduction. A suitable cost function is set to calculate the cost of the flyable area in the histogram based on the minimum cost principle. The best flight point for the aircraft is determined by its ability to avoid obstacles and reach the target point. The 3D-VFH algorithm divides the space around the aircraft into multiple grids along the pitch angle and yaw angle dimensions, with each grid representing a possible forward direction for the aircraft.

This paper proposes improvements to the 3D-VFH algorithm by combining a target point coordinate transformation method using 3D coordinates with a sliding window optimization algorithm for detecting flight directions. To ensure accurate control of the UAV’s flight towards the target point, the target point coordinates are transformed into the UAV’s body coordinate system. To ensure the feasibility of the UAV’s flight direction, a 4 × 4 sliding window is used to detect the flight direction of the UAV in the 2D histogram. Compared to the 3 × 3 sliding window, the 4 × 4 sliding window samples more data points, which improves detection speed. Finally, a cost function is used to select the optimal flight path from $V_{\mathrm{safe}}$.

### 3.1. Point Cloud Processing and Coordinate Conversion

Preprocess Light Detection and Ranging (LiDAR) point cloud data using a straight through filter and retain data that satisfy $-r_{\max}<x<r_{\max}\;\mathrm{and}\;-r_{\max}<y<r_{\max}\;\mathrm{and}\;-r_{\max}<z<r_{\max}$ ($r_{\max}$ is the maximum radius of the point cloud).

Due to the large volume of point cloud data, it is necessary to convert it to a OctoMap shaped data format with a smaller data size. In order to control the UAV to fly in the direction of the target point from the perspective of the UAV, the current position of the target point is required. Therefore, before calling the 3D-VFH algorithm, the coordinates of the target point in the map coordinate system need to be transformed to the UAV body coordinate system using the current positioning data of the UAV.

During the flight of the UAV, the deviation of the body coordinate system in the XOY plane does not affect the position of the target point. Therefore, before performing coordinate transformation, it is necessary to filter the quaternion $\left(w,x,y,z\right)$ representing the UAV attitude in the UAV positioning data and only retain the tilt angle Yaw rotated around the Z axis. The calculation formula is as follows:

(1) $$\mathrm{Yaw}=\tan^{-1}\frac{2\cdot \left(w\cdot z+x\cdot y\right)}{1-2\cdot \left(z^{2}+y^{2}\right)}$$

**Figure 1:**
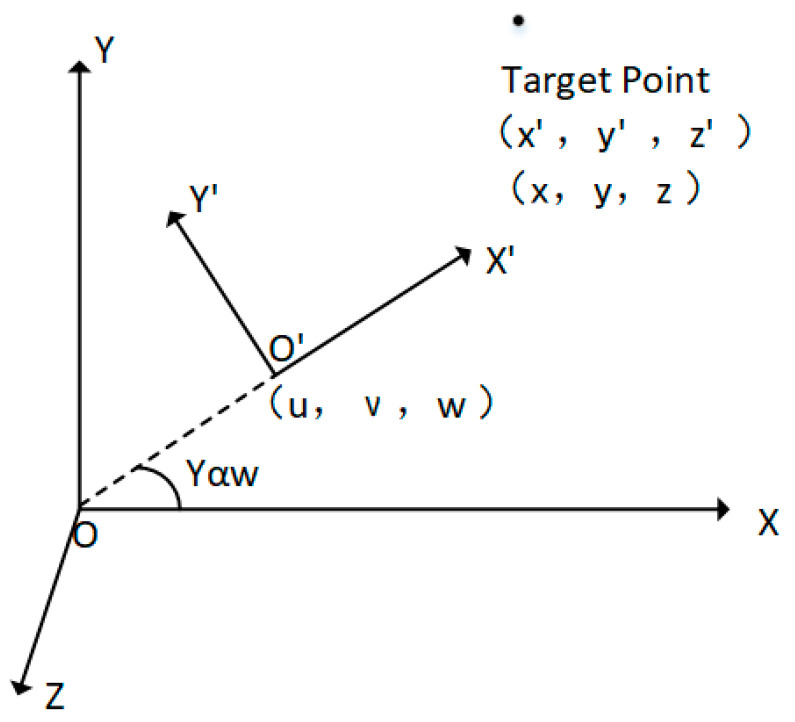
Schematic diagram of target point coordinate conversion.

Figure 1 is the schematic diagram of coordinate conversion. Let the map coordinate system be XYZ, and the UAV body coordinate system be $X^{\prime },Y^{\prime },Z^{\prime }$. The included angle of the two coordinate systems on the XOY plane is Yaw, the coordinates of the UAV under the map coordinate system are $\left(u,\;v,\;w\right)$, and the coordinates of the flight target point under the map coordinate system are $\left(x,y,z\right)$. The coordinates of the flight target point under the UAV coordinate system $\left(x^{\prime },y^{\prime },z^{\prime }\right)$ need to be calculated from the above data.

In the three-dimensional coordinate system, the UAV coordinate system and the map coordinate system do not share the same origin, so the coordinate system needs to be translated first and then rotated. The coordinate Formula (2) is as follows:

(2) $$\left[\begin{array}{c} x^{\prime }\\ y^{\prime }\\ z^{\prime } \end{array}\right]=\left[\begin{array}{ccc} \cos\left(\mathrm{Yaw}\right) & \sin\left(\mathrm{Yaw}\right) & 0\\ -\sin\left(\mathrm{Yaw}\right) & \cos\left(\mathrm{Yaw}\right) & 0\\ 0 & 0 & 1 \end{array}\right]\cdot \left(\left[\begin{array}{c} x\\ y\\ z \end{array}\right]+\left[\begin{array}{c} -u\\ -\nu \\ -w \end{array}\right]\right)$$

Finally, rewrite in the form of homogeneous coordinates and convert the flight target point from the coordinates under the map coordinate system to the coordinates under the UAV body coordinate system, as shown in Formula (3):

(3) $${\left[x^{\prime }\;y^{\prime }\;z^{\prime }\;1\right]}^{T}=\left[\begin{array}{cc} S_{z} & T\\ 0 & 1 \end{array}\right]\cdot {\left[x\;y\;z\;1\right]}^{T}$$

The transformation matrix is $S_{z}$, when it rotates counterclockwise around the Z axis. T represents the translation transformation matrix, which is calculated by the following Formula (4):

(4) $$T=S_{z}\cdot \left[\begin{array}{c} -u\\ -\nu \\ -w \end{array}\right]$$

### 3.2. Calculate the Weight of Obstacle Avoidance Route

**Figure 2:**
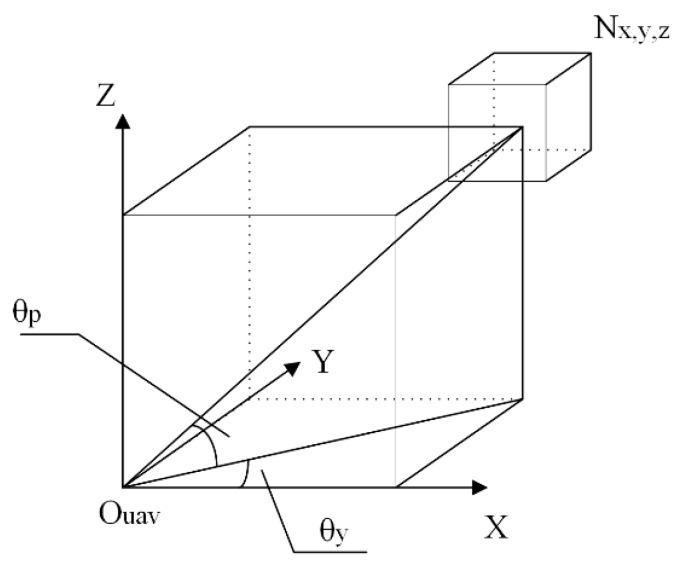
Schematic diagram of octoMap node angle calculation.

To calculate the obstacle avoidance weight, all OctoMap nodes need to be calculated one by one (Figure 2). Set the current OctoMap node to be $N_{x,y,z}$, whose subscripts indicate that the central coordinates of the corresponding nodes are (x,y,z), as shown in Formula (5).

(5) $$\theta_{y\;}=\tan^{-1}\frac{y}{x}\theta_{p\;}=\tan^{-1}\frac{z}{\sqrt{x^{2}+y^{2}}}$$

**Figure 3:**
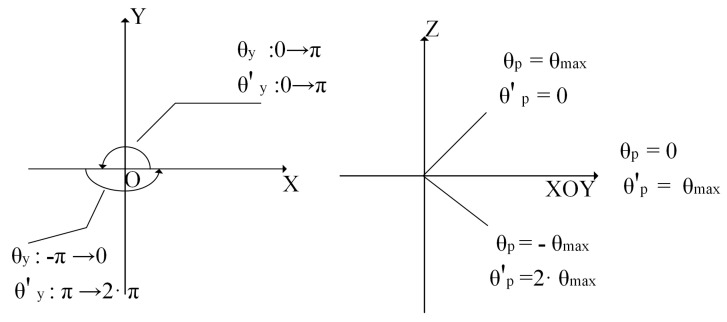
$\theta_{y\;}$ and $\theta_{p\;}$ conversion of value range.

As shown in Figure 3, the ${\theta^{\prime }}_{y}$ and ${\theta^{\prime }}_{p}$ obtained from the conversion are made continuous, and according to the angle step of each grid of histogram α,α represents the edge length of the two-dimensional histogram angle grid, α = 4, the subscripts $\left(i_{N},j_{N}\right)$ are calculated as follows in Formula (6):

(6) $$i_{N}=\left\{\begin{array}{c} \lfloor \frac{1}{\alpha }\cdot \theta_{y}\rfloor \left(\theta_{y\;}\geq 0\right)\\ \lfloor \frac{1}{\alpha }\cdot \left(2\pi +\theta_{y}\right)\rfloor \left(\theta_{y\;}<0\right) \end{array}\right.j_{N}\lfloor \frac{\theta_{\max}-\theta_{p}}{\alpha }\rfloor$$

Via node $N_{x,y,\;z}$ distance from the center of the vehicle *O_uav_* ${\;d}_{N}$ and safety range $r_{s+n}$, calculate $N_{x,y,z}$, the maximum angle that can affect φ and the number of meshes affected in one direction n, see Formula (7):

(7) $$\varphi =\tan^{-1}\frac{r_{s+n}}{d_{N}}\;\;n=\lfloor \frac{\varphi }{\alpha }\rfloor$$

**Figure 4:**
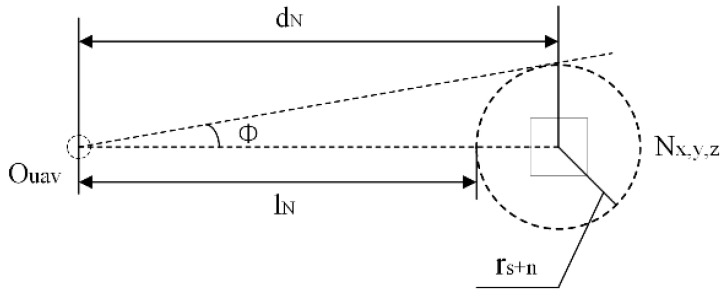
Schematic diagram of the node influence angle.

The vehicle safety radius $r_{s}$ and the maximum radius of the current node $r_{N}$ are used to get the safety range $r_{s+n}$, from UAV center *O_uav_* to node $N_{x,y,z}$. The distance of the safety range is $l_{N}=d_{N}-r_{s+n}$. As shown in Figure 4.

The weight of the node $N_{x,y,z}$ depends on the size of the node’s occupancy $o_{N}$ and $l_{N}$. $H_{i,j}$ represents the histogram grid weight with subscripts i,j, and the calculation Formula (8) is as follows:

(8) $$H_{i,j}=\left\{\begin{array}{c} H_{i,j}+{\left(o_{N}\right)}^{2}\left(a-b\cdot l_{N}\right)\;\;\;\;\;\;i\in \left[i_{N}-n,i_{N}+n\right]\\ \;\;\;\;\;\;\;\;\;\;\;\;\;\;\;\;\;\mathrm{and}\;\;j\in \left[j_{N}-n,j_{N}-n\right]\\ H_{i,j}\;\;\;\;\;\;\;\;\;\;\;\;\;\;\;\;\;\;\;\;\;\;\;\;\;\;\;\mathrm{otherwise} \end{array}\right.$$

The parameters a and b are two parameters with a relative relationship. Their specific values are not important. The relative relationship between a and b is determined by the following Formula (9):

(9) $$a-b{\left(\frac{2\cdot r_{\max}-1}{2}\right)}^{2}=1$$

### 3.3. Adaptive Selection of an Obstacle Avoidance Path

**Figure 5:**
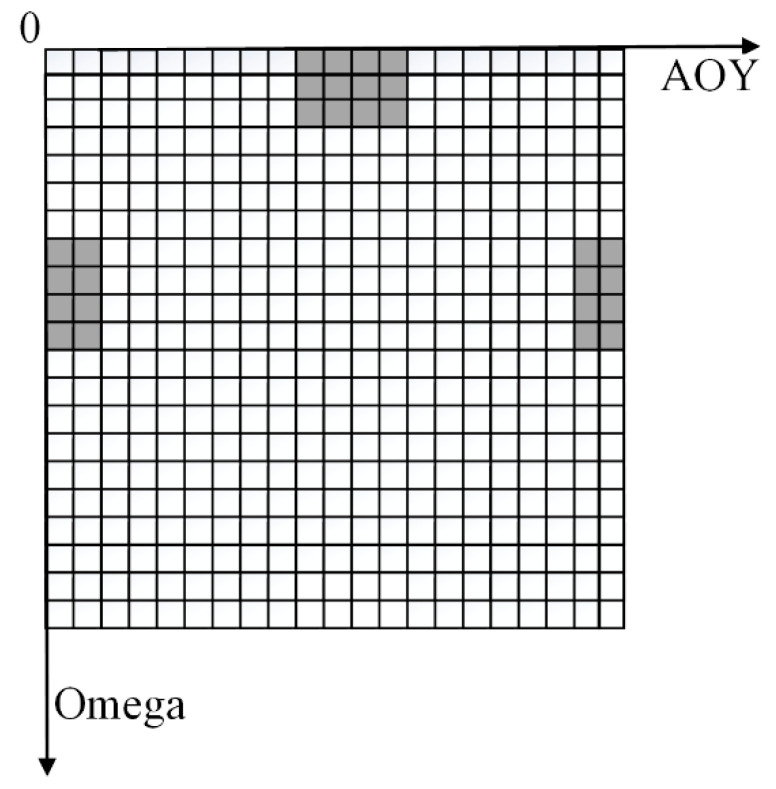
Schematic diagram of a sliding window.

Due to the small angle range represented by each grid, multiple consecutive safe grids are required to ensure that the corresponding direction is a feasible direction for the UAV to fly. Therefore, this paper uses a sliding window method to detect the candidate flight directions in the 2D histogram of the UAV’s flight. As shown in Figure 5.

Firstly, assuming a sliding window size of 4 × 4 and a horizontal field of view of 360° for the LiDAR, the sliding window extends to the same height on the opposite side of the histogram, as there are no blind spots at the left and right boundaries. Since the LiDAR’s vertical field of view is only 30°, there is no connection between the upper and lower parts of the generated 2D histogram, and the parts beyond the histogram range can be ignored.

Secondly, a sliding window is used to detect all directions in the histogram, and the set $V_{\mathrm{safe}}$ of all feasible candidate directions is generated by finding the directions where all values in the sliding window are 0. The paper represents the candidate directions in the form of unit direction vectors. $\vec{v_{\mathrm{best}}}$ represents the direction vector for the best forward direction of the drone in the current set $V_{\mathrm{safe}}$.

Finally, it is necessary to select the optimal forward direction for the current UAV from the set of candidate directions $V_{\mathrm{safe}}$ using a cost function. Let one of the candidate directions be denoted as $\vec{v_{i}}$. Considering that the LiDAR used in this paper has the ability to detect in a 360° direction, it is not necessary to rotate the UAV when controlling it, and the direction of the UAV’s head does not need to be taken into account. Therefore, the cost function used in this paper is shown in Formula (10):

(10) $${\mathrm{score}}_{i}=w_{1}\cdot \Delta \left(\vec{v_{i}},\;\vec{v_{\mathrm{goal}}}\right)+w_{2}\cdot \Delta \left(\vec{v_{i}},\;\vec{v_{\mathrm{pre}}}\right)$$

Set the direction selected by the previous algorithm to $\vec{v_{\mathrm{pre}}}$. The formula for calculating the angle difference between two direction vectors is shown in Formula (11):

(11) $$\Delta (\vec{v_{1}},\;\vec{v_{2}})=\cos^{-1}\frac{x_{1}\cdot x_{2}+y_{1}\cdot y_{2}+z_{1}\cdot z_{2}}{\sqrt{x_{1}^{2}+y_{1}^{2}+z_{1}^{2}}\cdot \sqrt{x_{2}^{2}+y_{2}^{2}+z_{2}^{2}}}$$

The cost function eliminates the consideration of the heading direction and sets the weights of the remaining two factors to w_1_ = 5, w_2_ = 3. After analysis, the weight values selected for these two weights are the optimal values. The best forward direction vector $\vec{v_{\mathrm{best}}}$ for the current UAV is selected through the cost function V_safe_. The UAV’s flight speed is determined by two factors: the distance between the UAV and the target point and the angle between the flight direction and the target point direction Δ($\vec{v_{i}}$, $\vec{v_{\mathrm{goal}}}$). To determine whether the UAV is avoiding obstacles, Δ($\vec{v_{i}}$, $\vec{v_{\mathrm{goal}}}$) is compared with the direct histogram direction step α. If the UAV is avoiding obstacles, the UAV speed is set to the preset obstacle avoidance speed. If the UAV is not avoiding obstacles, the UAV speed is set to the corresponding range of preset speeds based on the distance range of ${\;d}_{\mathrm{goal}}$.

## 4. Simulation Analysis and System Testing

Experiment with the research method, analyze its application performance, and understand the attitude stability and altitude stability of UAV flight under the improved 3D-VFH algorithm.

### 4.1. Flight Test Platform

#### 4.1.1. Hardware Experiment Platform

**Figure 6:**
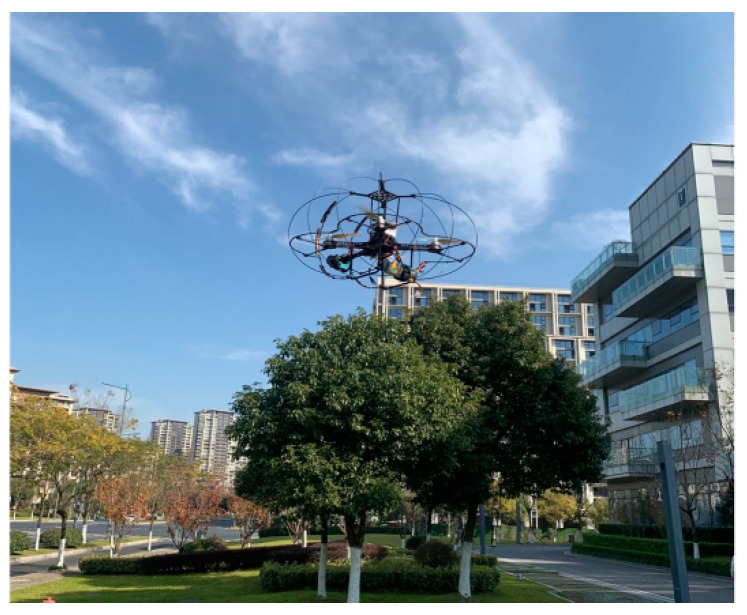
Experiment platform.

Flight tests were conducted on a closed-loop autonomous flight test platform shown in Figure 6. The experimental platform for the UAV mainly consists of a Raspberry Pi 3B+, a Pixhawk flight control board, a T265 camera, VLP-16 LiDAR, brushless motors, and a power module [34]. The Raspberry Pi 3B+ onboard computer runs the Ubuntu 18.04 system, which processes the position data collected by the T265 visual positioning and the laser radar in real-time. The position estimation is then performed by fusing the IMU position information from the flight control board. Finally, after receiving the control commands from the onboard computer, the flight control board controls the attitude and position of the UAV. Adopting a four-wing drone with a flight speed of 3 m/s. The safety radius of the drone is 1 m, and the maximum range of environmental information perception is 7 m.

#### 4.1.2. Software Operating System

The software operating system is mainly responsible for inter-system communication, sensor data acquisition, position state estimation, and position control.

**Figure 7:**
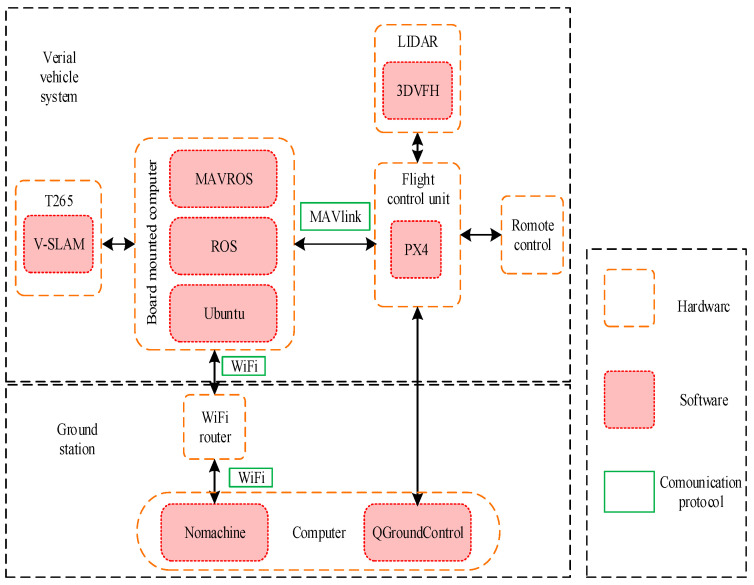
The UAV software system scheme.

The MavlinkRobot Operating System (MAVROS) communication protocol is an extremely lightweight message marshalling library designed for micro-UAVs. Based on the Robot Operating System (ROS) architecture, this system utilizes MAVROS for subscribing and publishing information within the onboard computer’s internal nodes and for communication between the onboard computer, the Raspberry Pi 3B+, and the Pixhawk flight control board. The control, status, and position information of the UAV can all be compiled into MAVROS data packets, which are transmitted between the ground control station and the UAV. Figure 7 depicts the software system solution for autonomous flight of the multi-rotor unmanned aerial vehicle in a sealed environment.

For the localization of the UAV in a closed environment, a T265 visual camera was utilized, which has an inbuilt Simultaneous Localization and Mapping (SLAM) module and can provide real-time position information to the onboard computer for precise localization. This was achieved using the px4-command open-source ROS package. The px4-pos-estimator node subscribed to the T265 visual camera and published position information, which was then converted to appropriate coordinates and published to the flight controller along with the corresponding yaw angle. Finally, the Move node issued commands for precise control of the UAV, such as position control, speed control, takeoff, and landing.

### 4.2. Autonomous Flight Test

**Figure 8:**
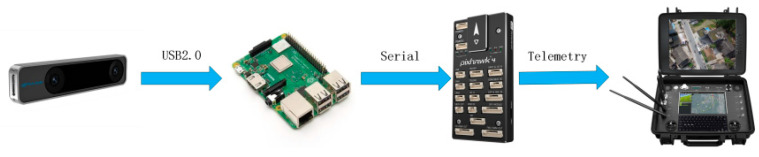
Technical scheme of autonomous flight tests.

**Figure 9:**
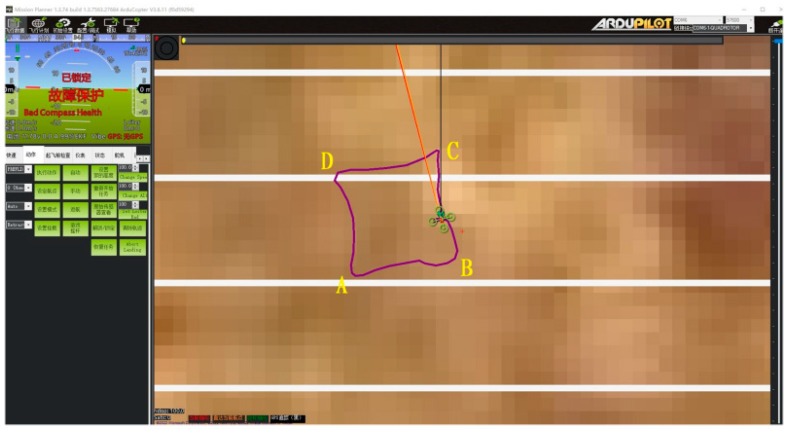
Autonomous flight waypoint planning.

To verify the autonomous flight stability of the UAV based on binocular vision, the autonomous flight test plan is shown in Figure 8. Considering the specific test conditions, the test plan for autonomous flight in the confined environment is designed as follows: The closed quadrilateral is composed of four task points: A, B, C, and D. Point A is set as the hovering point after takeoff at a height of 1.5 m. The side length of the quadrilateral flight path is set to 1 m, and the relative positions between the remaining task points and the starting point are shown in Figure 9.

Experimental Procedure: Prior to executing the quadrilateral flight mission, the UAV will take off and hover for a certain period of time. Subsequently, the UAV will perform autonomous flight on a quadrilateral trajectory, passing through the four edges AB, BC, CD, and DA, and finally end the mission with an autonomous landing at point A.

#### 4.2.1. Attitude Stability Test

**Figure 10:**
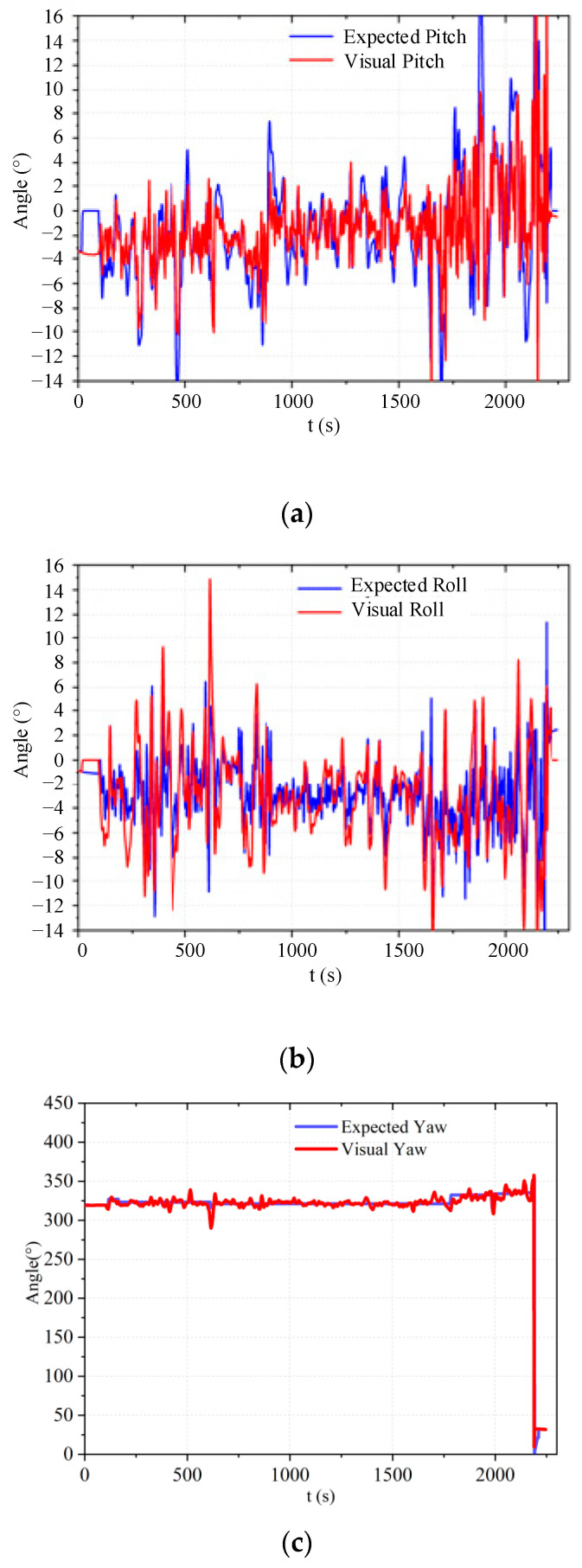
Attitude angle stability curve.

After the experiment, the real flight trajectory was obtained by analyzing the UAV flight log with ground station software, as shown in Figure 9. During the entire experiment, the collaborative computer running the task management system was able to control the UAV to take off and land autonomously and perform straight-line flights between multiple task points according to the experimental plan, and all tasks were executed as expected. Among them, based on the confined environment, the autonomous flight stability of the aircraft was tested, and the attitude stability curve was further tested through the flight log, as shown in Figure 10.

The following results were obtained from the flights, as depicted in Figure 10.

1. Figure 10a shows the actual and desired pitch angle curves of a quadrotor UAV. It can be seen from the figure that the maximum positioning error of the binocular vision system in a closed environment is between 4° and 6°, which satisfies the positioning requirements in a closed environment. The quadrotor UAV performs well in hover mode for forward and backward flight.
2. Figure 10b shows the actual and desired roll angle curves of a quadrotor UAV. It can be seen from the figure that the maximum positioning error of the binocular vision system in a closed environment is between 4° and 7°, which satisfies the positioning requirements in a closed environment. The quadrotor UAV performs well in hover mode for left and right flights.
3. Figure 10c shows the actual and desired yaw angle curves of a quadrotor UAV. It can be seen from the figure that the positioning accuracy of the binocular vision system for the yaw angle satisfies the positioning requirements in a closed environment. The quadrotor UAV performs well in hover mode.

#### 4.2.2. Flight Altitude Stability Test

**Figure 11:**
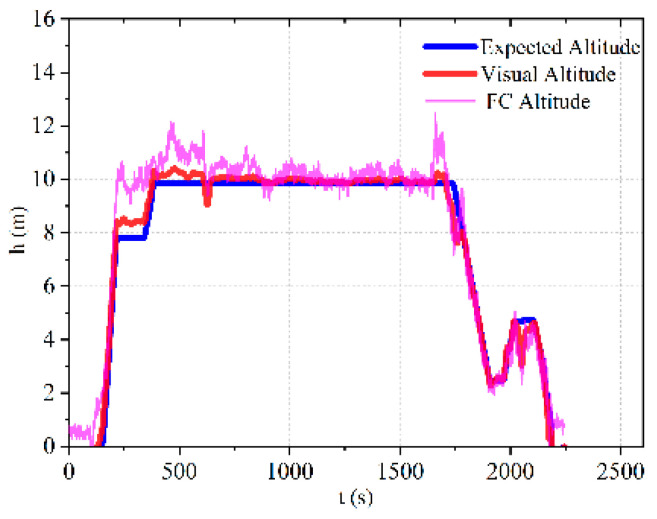
Flight altitude curve.

As shown in Figure 11, an experimental verification was conducted using flight log data to analyze the stability of flight altitude during fixed-point hovering of a quadrotor UAV.

According to Figure 11, in the fixed-point hovering mode, the stability curve of the actual visual height is consistent with the expected height, with a maximum error of about 0.3 m. The measured height by the barometer is subject to some fluctuation compared to the actual height. Through experiments, it has been proven that using binocular vision technology to achieve stable positioning of the UAV in the confined environment is feasible, and the reliability of autonomous flight of the UAV in the confined environment through planned waypoint trajectories has been demonstrated.

In summary, the overlap and follow-up of the actual flight trajectory and the planned trajectory of the UAV are excellent. The T265 binocular camera and the flight control system’s estimated position are roughly the same. Through analysis, it has been found that the UAV can accurately complete autonomous flight tasks in a closed environment, including active obstacle avoidance.

### 4.3. Outdoor Attitude Stability Test

**Figure 12:**
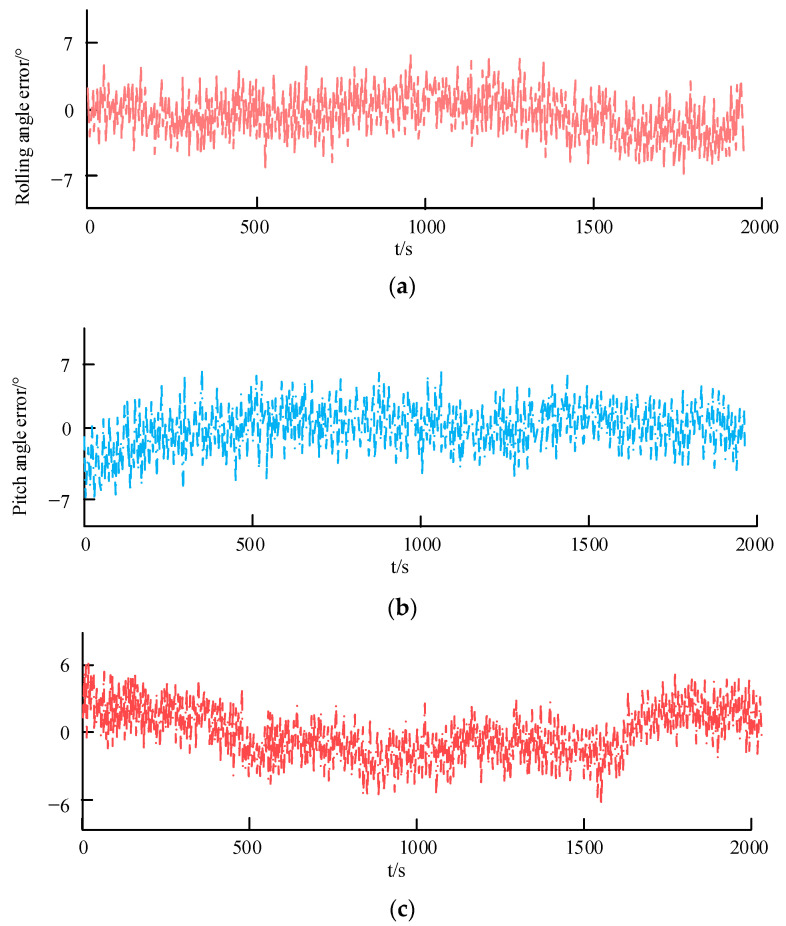
Outdoor attitude angle positioning error.

The attitude stability under general outdoor conditions is analyzed, and the actual and expected attitude angle error of the quadrotor UAV is studied, as shown in Figure 12.

According to Figure 12,

(1) Figure 12a shows the error curve of the actual roll angle and the expected roll angle of a quadrotor UAV. From the figure, it can be seen that the error of rolling angle positioning accuracy is within 7°, which meets the positioning requirements in outdoor environments.
(2) Figure 12b shows the error curve of the actual and expected pitch angles of the quadrotor UAV. From the figure, it can be seen that the error of rolling angle positioning accuracy is within 7°, which meets the positioning requirements in outdoor environments.
(3) Figure 12c shows the error curve of the actual yaw angle and the expected yaw angle of the quadrotor UAV. From the figure, it can be seen that the error of rolling angle positioning accuracy is within 6°, which meets the positioning requirements in outdoor environments.

### 4.4. Autonomous Obstacle Avoidance Test

**Figure 13:**
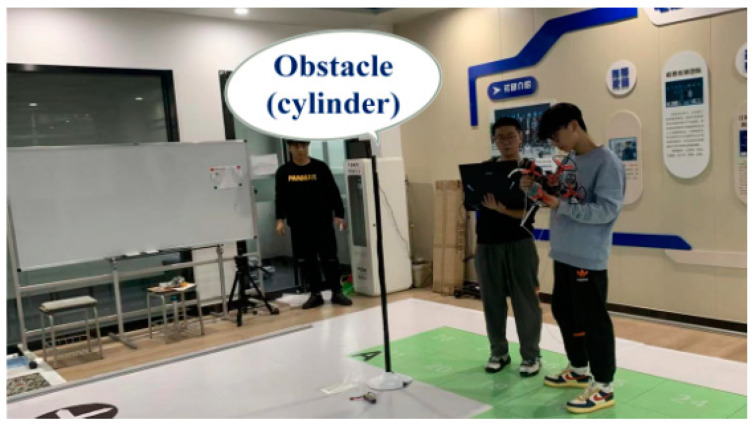
Indoor obstacle avoidance test scenario.

Due to the presence of many uncertain obstacles in the flying environment of UAVs, the obstacle detection of LIDAR may have errors due to the size characteristics of the uncertain obstacles, which can lead to UAV collisions and malfunctions. Therefore, to verify the autonomous obstacle avoidance stability of the quadrotor UAV, a cylindrical obstacle (H = 170 cm, R = 3 cm) was placed on the autonomous flight waypoint trajectory in a confined environment. The experimental scheme was to take off from the starting point and fly autonomously along the route, automatically avoiding obstacles encountered, and finally landing at the target point. The experimental environment is shown in Figure 13.

**Figure 14:**
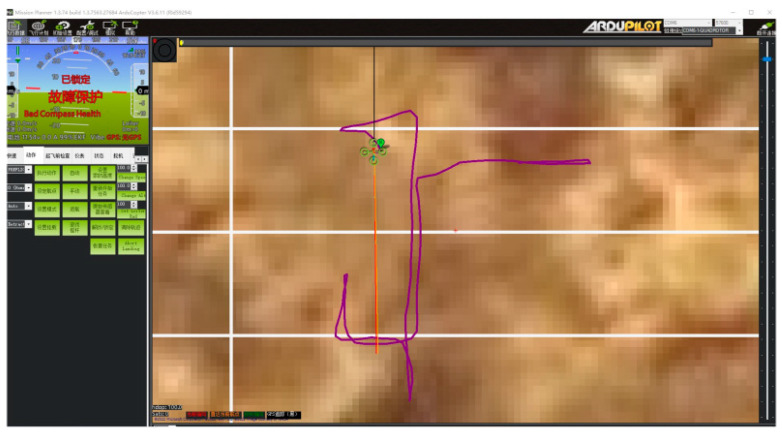
Path of the UAV obstacle avoidance waypoint.

**Figure 15:**
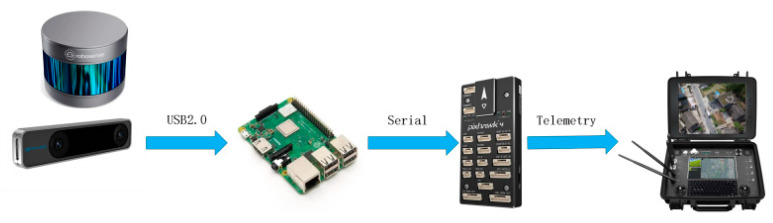
Technical scheme of autonomous obstacle avoidance.

In this test, the path given by global path planning did not avoid dynamic obstacles on the map. Dynamic obstacles refer to moving obstacles that have not been analyzed, which are equivalent to obstacles that do not exist on the global map. The UAV needs to use the radar carried onboard to avoid obstacles and reach the target point. According to the experimental requirements, the waypoint route of the UAV is set as shown in Figure 14, and the autonomous obstacle avoidance technology scheme is shown in Figure 15.

#### 4.4.1. Autonomous Obstacle Avoidance Test

**Figure 16:**
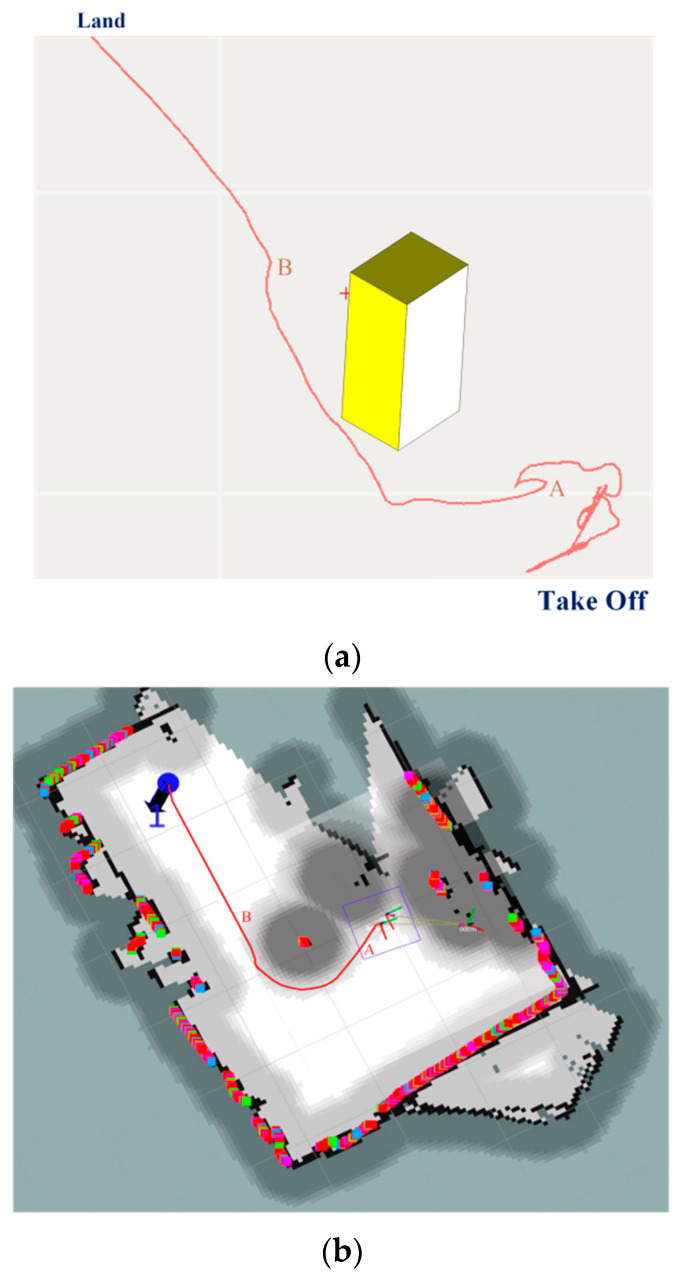
(**a**,**b**) Obstacle avoidance trajectory of the UAV.

In the experiment, a takeoff point (A), a target point (B), and an obstacle (a pillar) were set in the flight path, and two flight path points were set to simulate obstacle avoidance in the global path. The requirement was for the aircraft to actively avoid the obstacle (pillar) when flying from the takeoff point (A) to the target point (B) in order to test the local obstacle avoidance flight function. The experimental results are shown in Figure 16.

According to Figure 16, the UAV can actively and effectively avoid dynamic obstacles present in the global path during flight and correctly reach the target points in the global path in the planned order of waypoints. This proves the correctness of the local obstacle avoidance flight control function. The algorithm detects obstacles in 0.5 s.

#### 4.4.2. Height Stability Test

The autonomous obstacle avoidance function of the UAVis achieved by changing the yaw angle, and the stability of the UAV’s altitude is crucial for realizing autonomous obstacle avoidance. Therefore, research on the stability of flight altitude is of great importance.

**Figure 17:**
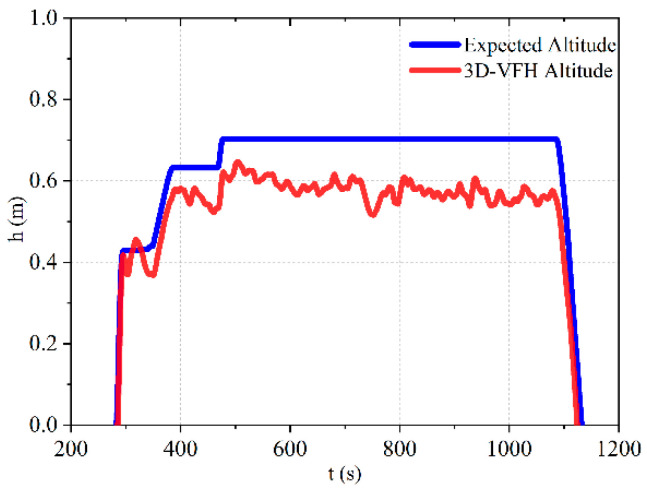
Autonomous obstacle avoidance flight altitude curve.

According to Figure 17, in a confined environment, the expected and actual height curves of autonomous obstacle avoidance using visual fixed-point hovering and laser radar methods maintain a similar trend, with a maximum error in actual flight height of around 0.1 m and the overall height curve of flight remaining at 0.6 m. Through experimental analysis, the feasibility of implementing autonomous obstacle avoidance for quadrotor UAVs in confined environments using LiDAR has been verified, and the reliability of implementing local obstacle avoidance for UAVs in confined environments using the 3D-VFH algorithm has been demonstrated.

**Figure 18:**
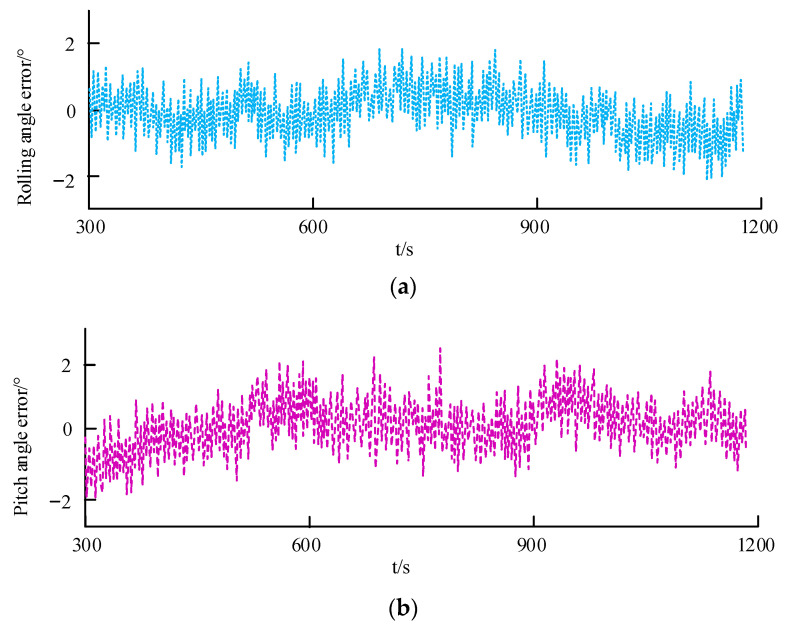
Rolling and pitch angle errors.

According to Figure 18,

(1) Figure 18a shows the error curves of the actual and expected rolling angles for autonomous obstacle avoidance using visual fixed-point hovering and LiDAR methods. From the figure, it can be seen that the error of the rolling angle is within 2°, which meets the stability requirements for obstacle avoidance in closed environments.
(2) Figure 18b shows the error curves of the actual and expected pitch angles for autonomous obstacle avoidance using visual fixed-point hovering and LiDAR methods. From the figure, it can be seen that the error of the rolling angle is within 2°, which meets the stability requirements for obstacle avoidance in closed environments.

#### 4.4.3. Attitude Stability Test

In the process of autonomous obstacle avoidance, attitude stability is a prerequisite for the UAV to complete local obstacle avoidance. Therefore, analyzing the attitude stability of the UAV during local obstacle avoidance has important practical significance.

**Figure 19:**
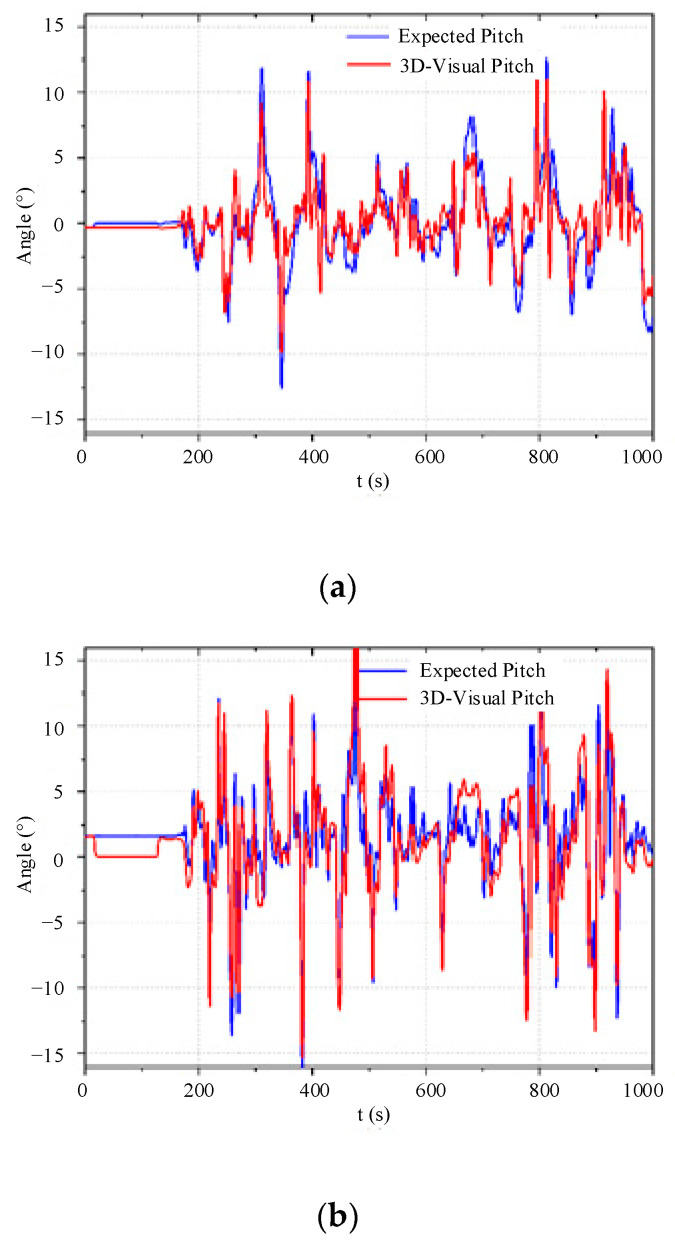

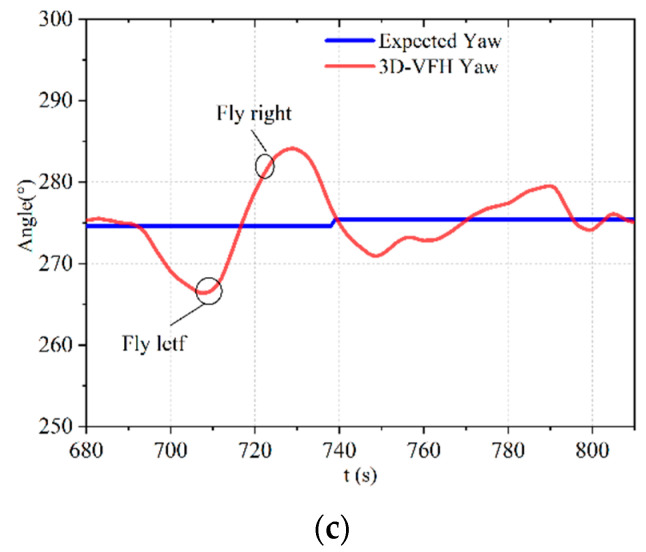
Visual obstacle avoidance attitude stability curve.

According to Figure 19,

(1) Figure 19a shows the actual pitch angle and expected pitch angle curves of the quadrotor UAV during autonomous obstacle avoidance. It can be observed from the figure that the maximum error in pitch angle precision is between 2° and 3°, which satisfies the stability requirements for obstacle avoidance in the confined environment and ensures the forward and backward motion functions during obstacle avoidance.
(2) Figure 19b shows the actual roll angle and expected roll angle curves of the quadrotor UAV during autonomous obstacle avoidance. It can be seen from the figure that the maximum error in roll angle precision is between 2° and 3°, which satisfies the stability requirements for obstacle avoidance in the confined environment and ensures the left and right motion functions during obstacle avoidance.
(3) Figure 19c shows the actual yaw angle and expected yaw angle curves of the quadrotor UAV during autonomous obstacle avoidance. When combined with the obstacle avoidance trajectory shown in Figure 15, it can be observed that the UAV first flies left and then right to achieve local obstacle avoidance. The yaw angle curve is consistent with the actual obstacle avoidance trajectory, and the yaw angle precision satisfies the stability requirements for obstacle avoidance in the confined environment, ensuring the yaw motion function during obstacle avoidance.

In method (1), Lindqvist B et al. used the nonlinear model predictive control (NMPC) method to achieve dynamic obstacle avoidance of UAVs in order to enable UAVs to achieve effective obstacle avoidance [35]. The drone trajectory type of method (1) is limited, while the research method takes into account the indoor and outdoor environment with a good autonomous obstacle avoidance function and better adaptability. In method (2), Wang C et al. utilized relevant 3D robot navigation algorithms to achieve the designated endpoint while avoiding obstacles in unmanned aerial vehicles (UAVs) based on non-holonomic robots [36]. This method eliminates the impact of unpredictable uncertainty and achieves safe flight, but it significantly increases the complexity and difficulty of the task. The research method has higher accuracy in controlling the position of drones under binocular vision and can accurately control drones. In method (3), Kownacki C et al. proposed a multidimensional repulsive potential field that considers the relative position of non-holonomic drones. The repulsive force is related to the distance from the UAV to the line representing the direction of obstacle movement [37]. Although the numerical simulation of this method is effective, it lacks verification in real environments. However, the research method can achieve effective flight in real environments, and the effects of automatic flight and obstacle avoidance are both good. Therefore, compared to methods (1) to (3), the advantages of research methods are more obvious. However, there are also certain shortcomings in the research methods. The obstacle avoidance effect on movable obstacles has not been tested, and a certain amount of random obstacles should be added, and the algorithm should be modified based on the obstacle avoidance effect.

## 5. Prospects

A research paper on autonomous flight and active obstacle avoidance of the quadrotor UAV has demonstrated that the quadrotor UAV can guide a flying vehicle to autonomously fly along a global path while avoiding unknown obstacles on a static map. The paper has proven that the 3D-VFH algorithm can effectively guide the UAV to avoid dynamic obstacles that were not considered in the global path. However, the paper did not consider the situation where the target point overlaps with a dynamic obstacle when implementing local obstacle avoidance flight. As a result, the UAV may not be able to reach the target point and may hover near the obstacle. From the three subgraphs in Figure 19, it can be seen that during the autonomous obstacle avoidance process, the attitude stability of the drone is good, and its maximum errors in pitch and rolling angles are very small, ranging from 2° to 3°. And its yaw angle also has high accuracy.

## 6. Summary

This paper proposes improvements to traditional methods for autonomous localization and local obstacle avoidance of quadrotor UAVs in confined environments. By incorporating polar coordinates into the target point coordinate system transformation, the algorithm’s real-time performance is improved. The paper then applies a sliding window algorithm for position estimation and obstacle avoidance control of the UAV’s flight path, compensating for the inaccuracies caused by dynamic obstacles. Based on this, the paper presents an improved 3D-VFH obstacle avoidance algorithm that enables autonomous global path planning and rapid avoidance of dynamic obstacles, providing important guidance for the precise autonomous flight and obstacle avoidance of quadrotor UAVs in enclosed spaces. The results show that in the stability test of an unmanned aerial vehicle’s automatic flight attitude, the maximum pitch angle positioning error of the binocular vision system in a closed environment is between 4° and 6°, which meets the positioning requirements in a closed environment. In the flight attitude altitude stability test, the maximum error is approximately 0.3 m. In outdoor attitude stability testing, the error in rolling angle positioning accuracy is within 7°. In autonomous obstacle avoidance stability detection, the error of the rolling angle is within 2°, which meets the stability requirements for obstacle avoidance in closed environments. The following conclusions were drawn:

(1) The paper validates that by introducing the improved 3D-VFH algorithm based on binocular vision and LiDAR for 3D navigation, the UAV can complete autonomous flight tasks in confined environments and effectively avoid dynamic obstacles.
(2) The sliding window algorithm is improved to select the optimal route for the UAV’s flight path, enabling the UAV to fly directly towards the target direction without changing the heading direction and making the local obstacle avoidance function more reliable.
(3) Height and attitude stability have a significant impact on the UAV’s autonomous flight and obstacle avoidance. The research in this paper ensures the safety of UAVs for automated inspections and promotes the digitization of smart cities.

## Data Availability

The data presented in this study are available in this article.
